# Choosing an effective food classification system for promoting healthy diets in Thailand: a comparative evaluation of three nutrient profiling-based food classification systems (government, WHO, and Healthier Choice Logo) and a food-processing-based food classification system (NOVA)

**DOI:** 10.3389/fnut.2023.1149813

**Published:** 2023-05-17

**Authors:** Sirinya Phulkerd, Sarah Dickie, Natjera Thongcharoenchupong, Sasinee Thapsuwan, Priscila Machado, Julie Woods, Ladda Mo-Suwan, Piyada Prasertsom, Chantana Ungchusak, Chiraporn Khitdee, Mark Lawrence

**Affiliations:** ^1^Institute for Population and Social Research, Mahidol University, Salaya, Thailand; ^2^Institute for Physical Activity and Nutrition, School of Exercise and Nutrition Sciences, Deakin University, Geelong, VIC, Australia; ^3^Faculty of Medicine, Prince of Songkla University, Songkhla, Thailand; ^4^Bureau of Dental Health, Department of Health, Ministry of Public Health, Mueang, Nonthaburi, Thailand; ^5^Thailand Healthy Lifestyle Plan, Thai Health Promotion Foundation, Bangkok, Thailand

**Keywords:** nutrient profiling, NOVA food classification system, ultra-processed foods, healthy diets, non-communicable diseases, Thailand

## Abstract

**Introduction:**

This study aimed to assess the nutritional quality of food and beverage products in Thailand by comparing four different food classification systems: the nutrient profiling-based food classification systems by the Department of Health (DOH), the WHO South-East Asia Region (WHO SEA), the Healthier Choice Logo (HCL), and the food-processing-based food classification system, NOVA.

**Methods:**

This study used secondary data from the Mintel Global New Products Database (*N* = 17,414). Food subgroups were classified differently based on these four systems. The DOH classified food products into three groups: Group A—healthy pass or meeting standard, Group B—not meeting the standard, and Group C—far below standard. The WHO SEA classified food products into two groups: marketing prohibited products and marketing permitted products. The HCL classified food products into two groups: eligible products for the logo; and ineligible products for the logo. The NOVA classified food products into four groups: unprocessed or minimally processed foods (MP), processed culinary ingredients (PCI), processed foods (P), and ultra-processed foods (UPF). Descriptive statistics (percentage and frequency) were used for analysis. Agreement analysis was conducted using Cohen’s kappa statistic between each pair of food classification systems.

**Results:**

Of the total sample that could be classified by any of the four classification systems (*n* = 10,486), the DOH, the WHO SEA and the HCL systems classified products as healthy (Group A, marketing permitted or eligible for HCL logo) at 10.4, 11.1, and 10.9%, respectively. Only 5.6% were classified as minimally processed foods using NOVA and 83.1% were ultra-processed foods (UPFs). Over 50% of products classified as healthy by the nutrient profiling systems were classified as UPF according to the NOVA system. Products that were eligible for the HCL had the highest proportion of UPF products (84.4%), followed by the Group A products (69.2%) and the WHO marketing-permitted products (65.0%).

**Conclusion:**

A hybrid food classification approach taking both nutrients and food processing into account is needed to comprehensively assess the nutritional quality of food and beverage products in Thailand.

## 1. Introduction

Thailand has achieved five of the 10 indicator targets for the prevention and control of non-communicable diseases (NCD) according to the World Health Organization (WHO)‘s Non-communicable Diseases Progress Monitor 2020 (1). Despite this achievement, Thailand’s progress in implementing policies to address the modifiable NCD risk factors remains insufficient. NCDs are a major cause of mortality in Thailand with major NCDs (cardiovascular diseases, cancers, diabetes, and chronic respiratory diseases) contributing to 14% of all premature deaths in 2014 (2). Overweight, obesity, and unhealthy diets remain the top five major risk factors for NCDs nationally (3).

Research over the past 5 years has demonstrated that diets high in ultra-processed foods (UPFs) are associated with a higher prevalence of obesity, diabetes, cardiovascular disease, and certain cancers (4–6). UPFs have poor nutritional profiles, in part, because they are often high in added sugars, salt, and fats (5), and also due to changes to the food structure (matrix) in which the bioactive compounds are present, the types of additives used, and a reduction in foods’ protective components (7). Over the past 20 years, dietary consumption patterns of the Thai population have shifted from a traditional diet (high in legumes, fruit, vegetables, and fish) to a more Westernized diet (ultra-processed, high in dietary energy, and relatively low in nutrients) (8, 9). Thai people consume much higher levels of sodium (3,636 mg per day) and added sugar (24.3 teaspoons or 97.3 g) (10) than recommended by the WHO for disease control, which should be no more than 2,000 milligrams (mg) of sodium per day, and six teaspoons or 25 grams of added sugar per day (11, 12). The National Food Consumption Behaviour Survey reports that Thai people regularly consume convenience or ready-to-eat foods (13). In 2017, over 50% of the Thai population regularly consumed sugar-sweetened beverages, ready-to-eat foods, and snacks. The sales values of UPFs, in particular, ready-to-eat meals and soft drinks, increased by 10.7 and 2.1%, respectively, from 2018 to 2019 (14).

Effective food policy actions such as food labeling for promoting healthy dietary patterns can benefit from a scientific method to assess the health potential of individual foods, i.e., foods that are components of a healthy diet and foods that are not necessary for a healthy diet (15). Over the past decade, two types of food classification systems have received attention in public health-related fields: nutrient profiling (15) and food-processing-based classifications (16). The WHO describes nutrient profiling as “*the science of categorizing foods according to their nutritional composition* (17).” Nutrient profiling models vary in complexity and detail based on a country’s context and their purpose (15). Food-processing-based classifications focus on assessing food products by level of processing to promote traditional diets based on minimally processed foods and freshly prepared dishes and meals (18). For example, NOVA classifies foods into four groups based on the extent and purpose of their industrial food processing: unprocessed or minimally processed foods, processed culinary ingredients, processed foods, and ultra-processed foods (UPFs) (7).

In Thailand, two nutrient profiling models have been developed to assess the nutritional quality of a given food based on the amount of certain nutrients it contains. First, the Nutrient Profiling Scoring Criterion developed by the Department of Health (DOH) at the Ministry of Public Health Thailand was mainly developed to guide food and drink provision and sale in preschools and schools (19). This model is based on the WHO Nutrient Profile Model for the South-East Asia Region (SEA) (20). The model is also being considered to determine which foods and beverages should be permitted for marketing to children.

The DOH is currently revising its nutrient profile model for better alignment with Thai recommended daily intakes (Thai RDI) (21). Another nutrient profile model used in Thailand, the Healthier Choice Logo (HCL) system, developed by the Institute of Nutrition, informs a voluntary front-of-pack label (22). The HCL uses “two overlapping leaves” and the name of the food category to indicate overall nutritional quality, with a food company being able to display the HCL logo if the product meets the nutrition criteria for the specific food category (22). It includes the same nutrients as DOH, except for B2, and the threshold score is lower. These two models have not yet been evaluated for their ability to differentiate UPFs from healthy foods and help shift diets back to healthier, minimally processed traditional diets. This is especially important as a food-processing-based classification system such as NOVA is yet to be applied to policies in Thailand.

This study aimed to assess the nutritional quality of packaged food and beverage products in Thailand by comparing four different food classification systems: the DOH’s nutrient profiling model, the WHO SEA’s nutrient profiling model, the HCL system, and the NOVA system, which was selected as an appropriate food-processing-based assessment tool for addressing the growing concern about the consumption of UPFs. This study intends to contribute to a better understanding of different food classification models and to support policymakers in selecting a robust, science-based model for promoting healthier food choices in Thailand.

## 2. Materials and methods

This is a comparative research study that used secondary data on nutrition information and ingredients of Thai food and beverage products.

### 2.1. Data collection

Data on food and beverage products released into the Thailand marketplace over the period 2015–2021 were extracted from the Mintel Global New Products Database (Mintel GNPD). The Mintel GNPD is an independent provider of market research with global coverage of key new products launched (23).

All data were downloaded from the Mintel GNPD (*n* = 41,145), and then all products with the following criteria (*n* = 23,731) were removed: products with no nutrient information (*n* = 18,805); duplicates—products with identical nutrition and ingredients, but may differ in packaging or different sized packaging (*n* = 1,250); and food and drink categories not required—non-food and alcoholic items, and special supplementary foods, e.g., meal replacements and sports supplements (*n* = 3,676). The final number of products for analysis was 17,414.

Food subgroups were classified differently based on the following food classification systems: nutrient profiling developed by the DOH (24), nutrient profiling proposed by WHO SEA (20), nutrient profiling developed for voluntary HCL (22), and NOVA classification system which classifies foods based on the type of processing (7). The classification was conducted by two researchers independently (SD and NT) and checked by other researchers (SP, ST, PM, and JW). As some schemes only included criteria for particular food categories or could only be classified with comprehensive packaging information (e.g., the ingredients list for NOVA) some foods in the whole set were excluded and, therefore, the sample size for each scheme differed. The sizes for each classification scheme are as follows: DOH (*n* = 12,750), WHO SEA (*n* = 17,030), HCL (*n* = 12,282), and NOVA (*n* = 17,058).

#### 2.1.1. Department of Health nutrient profiling

**Figure 1:**
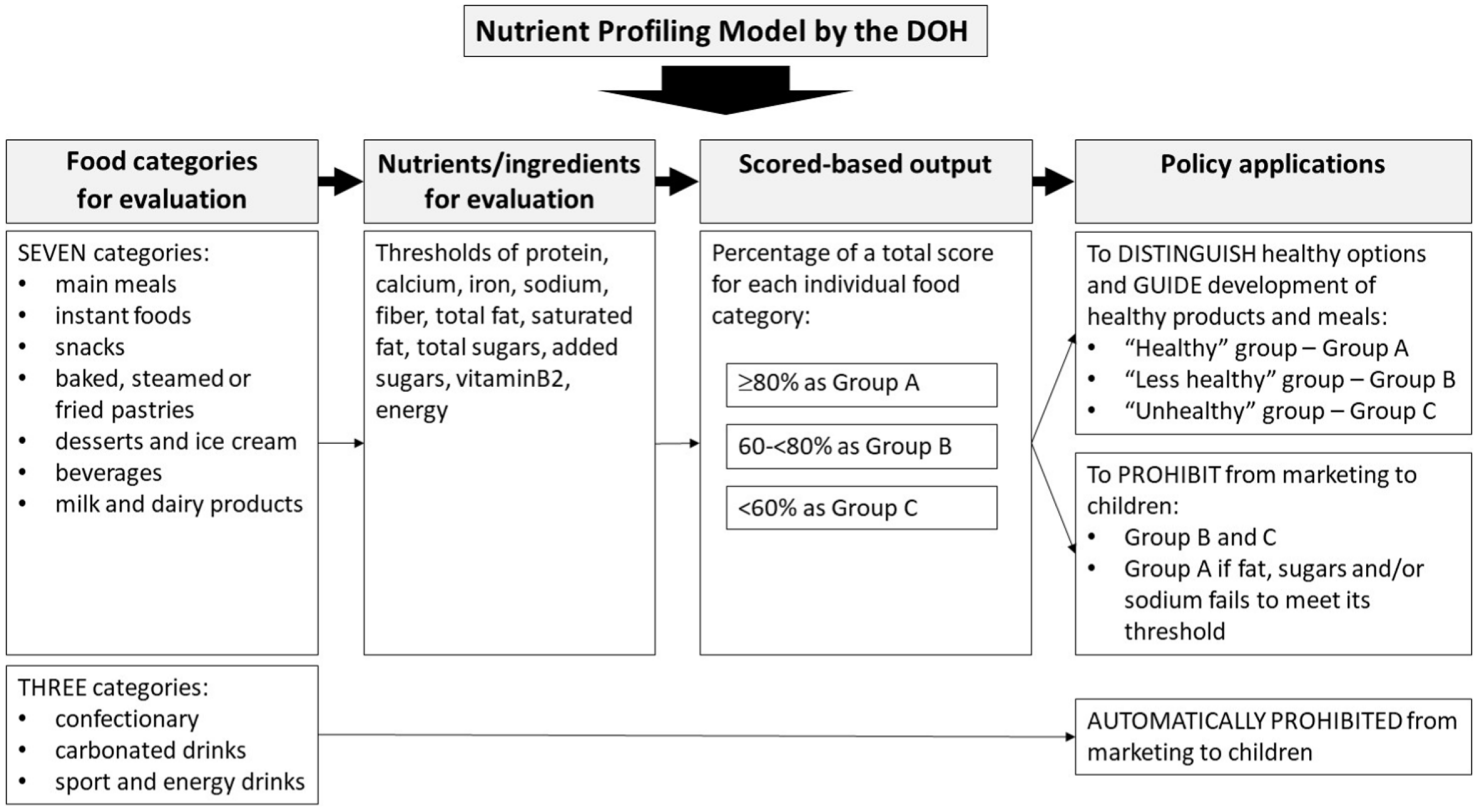
Summary of the nutrient profiling model developed by the Department of Health (24).

The DOH nutrient profiling model investigated in this study used the most recent version of the nutrient profiling criteria (15 October 2021) (24). Figure 1 shows the characteristics of the DOH model. This model classifies foods into seven categories, with nutrients assessed and thresholds applied differing by category. The DOH categories include freshly made dishes and meals; however, for the purpose of this study, only packaged products were assessed. Ten nutrients are included in the criteria—based on an expert consensus regarding nutrients of concern in the Thai population. The scores for the included nutrients are derived from the Thai RDI expressed on an energy basis and expert consensus. Foods with an overall score of ≥80%, 60–<80, and < 60% (out of a total score for each individual food category) are classified into Groups A, B, and C, respectively. Group A indicates healthy pass or meeting standard; Group B indicates not meeting the standard; and Group C indicates far below standard. As the DOH criteria have specific nutrient criteria for each food category, some food products are not eligible for evaluation. Food products that do not provide sufficient nutrient information were also excluded (*n* = 4,664).

#### 2.1.2. Who SEA’s nutrient profiling

The WHO SEA nutrient profile model was developed for Member States to use as a means of assessing eligibility for food marketing to children and for encouraging product reformulation for improving population nutrition (20). The WHO SEA model was partly based on the model developed by WHO Western Pacific Region (25) and thresholds are based on principles used in the population Nutrient Intake Goals of the Pan American Health Organization (PAHO) nutrient profile model (26).

The WHO SEA model classifies foods into 18 groups (Table 1) (20). However, non-packaged products that did not provide all required nutrient information were excluded from the analysis (*n* = 384).

**Table 1:** Percentage of sample in this study by food classification systems.

DOH model (*n* = 12,750)		WHO SEA model (*n* = 17,030)		HCL model (*n* = 12,282)		NOVA system (*n* = 17,058)	
Category	%	Category	%	Category	%	Category	%
Snacks	23.8	Beverages	23.6	Beverages	24.3	Ultra-processed foods	76.6
Beverages	23.6	Fine bakery wares	17.1	Snacks	23.6	Processed foods	14.6
Main meals	10.3	Composite foods	9.9	Bakery product	10.9	Unprocessed/minimally processed foods	6.1
Baked, steamed or fried pastries	14.6	Confectionery	8.7	Small meals	8.5	Processed culinary ingredients	2.7
Products prohibited from marketing	9.4	Ready-to-eat savories	7.7	Ready to eat meals	6.4		
Desserts and ice cream	8.3	Sauces dips, and dressings	6.7	Instant foods	6.0		
Instant foods	6.0	Processed fruits and vegetables	6.6	Dairy product	4.4		
Milk and dairy products	4.0	Processed meat, poultry, game, fish and fish products	3.8	Breakfast cereals	3.5		
		Frozen dairy based desserts and edible ices	4.1	Ice-cream	3.5		
		Cereals	3.4	Seasonings	3.1		
		Pasta and noodles and like products	2.9	Fat and oil	2.4		
		Fats and oils, and fat emulsions	2.7	Fish and other aquatic product	1.9		
		Bread and ordinary bakery wares	2.0	Breads	1.5		
		Cheese and analogs	0.4				
		Curded dairy based desserts	0.2				
		Solid-form soybean products	0.1				
		Fresh and frozen fruits and vegetables, and legumes	0.1				

DOH, the Department of Health; WHO SEA, the WHO South-East Asia Region; HCL, the Healthier Choice Logo; and NOVA, the food-processing-based food classification system.

The nutrient thresholds set in this system were based on principles used in the PAHO nutrient profile model (26), i.e., the population Nutrient Intake Goals (17). They include total fat, saturated fat, total sugars, added sugars, and sodium, and the threshold criteria depend on the food category. A product must not exceed (on a per 100 g/ml basis) any one of the relevant thresholds for that food product category if marketing is to be permitted (20).

#### 2.1.3. Healthier Choice Logo nutrient profiling

The HCL nutrient profiling model was developed to help consumers identify foods that are considered healthier choices. The HCL model is a voluntary front-of-pack nutrition label placed on packaged foods to promote healthier diets among Thai people (available on http://healthierlogo.com/) (22).

Foods are classified into 13 categories (Table 1). Nutrients and other components of foods selected for this model are based on policy-driven considerations for NCD prevention. The nutrients assessed differ for each food category. Nutrients included are protein, calcium, iron, sodium, fiber, total fat, saturated fat, total sugars, added sugars, and energy. Many food products (*n* = 5,132) were not eligible for inclusion in this study as they did not fall within the HCL category.

#### 2.1.4. NOVA classification system

According to Monteiro et al., food and beverage products are classified into one of four NOVA categories: unprocessed or minimally processed foods (MP), processed culinary ingredients (PCI), processed foods (P), and UPFs (7). Of particular interest, UPFs are identified by the presence of food substances of no (or rare) culinary use (e.g., protein isolates, high fructose corn syrup, maltodextrin, and modified starches) and/or classes of additives whose function is to make the final product palatable or more appealing (e.g., colorants, flavors, artificial sweeteners, emulsifiers, and thickeners) in the ingredients list (7). UPF products include snacks, drinks, ready meals, and many other product types formulated mostly or entirely from substances extracted from foods or derived from food constituents. These products are made possible by the use of many types of additives, including those that imitate or enhance the sensory qualities of whole foods and freshly made dishes.

For other groups, the MPs are identified by “unprocessed foods altered by industrial processes such as removal of inedible or unwanted parts, drying, crushing, grinding, fractioning, roasting, boiling, pasteurization, and refrigeration (6, 7). None of these processes add salt, sugar, oils or fats, or other food substances to the original food.” The PCI are substances “obtained directly from group 1 [MP] foods or from nature, like oils, butter, sugar, and salt.” The P are processed products “made by adding salt, sugar, or other PCI to group 1 [MP] foods, using preservation methods such as canning and bottling, and, in the case of bread and cheeses, using non-alcoholic fermentation.” Food products that did not provide or provided an incomplete list of ingredients were excluded from the analysis (*n* = 356).

#### 2.1.5. Fiber estimation

In this study, the fiber values were estimated for the DOH and HCL models because they are not required on the packaging (21, 22), and as such not available on many products. If the product belonged to a Mintel sub-category that typically does not contain fiber, the fiber value was recorded as zero. For other sub-categories, estimates were carried out using the following steps:

- Ingredient lists were scanned for fiber-containing ingredients, including industrially derived ingredients such as inulin, polydextrose, and oligofructose.
- For Mintel sub-categories containing similar-type products (e.g., sweet biscuits, pasta, and wet soups), an average value was calculated from all products declaring fiber values (excluding those recorded as zero). This average was then allocated to all products with fiber-containing ingredients with missing fiber values in that sub-category.
- For sub-categories that contained a more diverse range of products, products with missing fiber values were matched to similar products (with declared fiber).
- Products with fiber-containing ingredients in sub-categories that could not be averaged, or could not be matched to similar products, were conservatively allocated values of 1 g/100 g.

### 2.2. Statistical analysis

The analyses were conducted using STATA version 16. Descriptive statistics were calculated, including the frequency and percentage of total food items and by food groups in each food classification system. The number of products in each NOVA category according to the three nutrient profiling models was described.

Analysis was also conducted using Cohen’s kappa statistic to measure the degree of pairwise agreement between food classification systems (i.e., the pairwise agreement between systems for each individual product). Only the products that were classified by all of the four classification systems (the DOH, the WHO SEA, the HCL, and the NOVA systems) were used for this analysis (*n* = 10,486). The data were classified as healthy (Group A: marketing permitted, eligible for HCL logo, and non-UPF) and unhealthy (Groups B and C: marketing prohibited, not eligible for HCL logo, and UPF) according to each system prior to the analysis. Then, the kappa statistic for each pair of the four systems was performed. The values range from – 1 to 1 with 1 presenting complete agreement, 0 meaning no agreement, and a negative statistic implying the agreement is worse than random (27). Values in the range of 0.01–0.20 can be presented as “none to slight” agreement, “0.21–0.40” as fair agreement, “0.41–0.60” as moderate agreement, “0.61–0.80” as substantial agreement, and “0.81–1.00” as almost perfect agreement (28).

## 3. Results

### 3.1. Overall characteristics of the study sample

Of the total 17,414 products, there were 14,139 food products and 3,275 beverage products. The sample of products used for analysis varied by type of food classification systems: 12,750 products for the DOH model, 17,030 products for the WHO SEA model, 12,282 products for the HCL model, and 17,058 products for the NOVA system. Table 1 presents a breakdown of the sample by the proportion of products in each food category for each food classification system.

### 3.2. Evaluation of different food classification systems

Table 2 presents summary results of food classification based on the DOH, the WHO SEA, the HCL, and the NOVA systems.

**Table 2:** Percentage of packaged products that can be classified by all four classification systems.

DOH model (% of products)				WHO SEA model (% of products)			HCL model (% of products)			NOVA system (% of products)				
Food category	Group A	Group B	Group C	Food category	Marketing-prohibited group	Marketing-permitted group	Food category	Not eligible for the logo	Eligible for the logo	Food category	MP	PCI	P	UPF
Snacks	7.6	20.4	72.0	Beverages	72.4	27.6	Beverages	88.9	11.1	Confectionery	0.2	0.0	5.0	94.8
Beverages	29.2	23.9	46.9	Fine bakery wares	99.8	0.2	Snacks	93.9	6.1	Fine bakery wares	0.2	0.0	11.1	88.7
Main meals	0.0	0.1	99.9	Composite foods	97.8	2.2	Bakery products	99.9	0.1	Bread and ordinary bakery wares	0.3	0.0	33.4	66.3
Baked, steamed or fried pastries	0.3	1.2	98.4	Confectionery	97.6	2.4	Small meals	100.0	0.0	Cereals	7.7	0.0	19.1	73.3
Desserts and ice cream	1.3	36.4	62.3	Ready-to-eat savories	96.0	4.0	Ready to eat meals	76.4	23.6	Ready-to-eat savories (savory snack foods)	4.5	0.0	18.4	77.1
Instant foods	1.3	4.9	93.8	Sauces dips, and dressings	95.3	4.7	Instant foods	71.0	29.0	Beverages	12.5	0.1	9.0	78.4
Milk and dairy products	0.0	0.6	99.4	Processed fruits and vegetables	72.8	27.2	Dairy products	63.6	36.4	Frozen dairy based desserts and edible ices	0.9	0.0	9.0	90.2
				Processed meat, poultry, game, fish and fish products	98.6	1.4	Breakfast cereals	82.5	17.5	Curded dairy based desserts	0.0	0.0	0.0	100.0
				Frozen dairy based desserts and edible ices	97.3	2.7	Ice-cream	77.6	22.4	Cheese and analogs	1.6	1.6	49.2	47.5
				Cereals	94.9	5.1	Seasonings	74.1	25.9	Composite foods (prepared foods)	0.5	0.0	8.0	91.5
				Pasta and noodles and like products	94.3	5.7	Fat and oil	86.2	13.8	Fats and oils, and fat emulsions	2.9	81.3	6.0	9.8
				Fats and oils, and fat emulsions	35.5	64.5	Fish and other aquatic products	81.9	18.1	Pasta and noodles and like products	4.4	0.0	7.3	88.3
				Bread and ordinary bakery wares	98.0	2.0	Breads	87.8	12.2	Processed meat, poultry, game, fish and fish products	7.6	0.2	15.9	76.3
				Cheese and analogs	90.2	9.8				Fresh and frozen fruits and vegetables, and legumes	80.0	0.0	6.7	13.3
				Curded dairy based desserts	65.7	34.3				Processed fruits and vegetables	17.4	0.0	42.9	39.7
				Solid-form soybean products	100.0	0.0				Solid-form soybean products	46.7	0.0	46.7	6.7
				Fresh and frozen fruits and vegetables, and legumes	20.0	80.0				Sauces dips, and dressings	3.1	3.2	31.6	62.0

DOH, the Department of Health; WHO SEA, the WHO South-East Asia Region; HCL, the Healthier Choice Logo; and NOVA, the food-processing-based food classification system; MP, unprocessed or minimally processed foods; PCI, processed culinary ingredients; P, processed foods; and UPF, ultra-processed food. Group A indicates healthy pass or meeting standard; Group B indicates not meeting standard; and Group C indicates far below standard.

For *the DOH model,* of 12,750 products, 1,202 products were automatically classified as unhealthy and prohibited from marketing (e.g., fizzy drinks and confectionery), leaving 11,548 that could be assessed into Groups A, B, and C. Of these, 9.4% were classified into Group A, 15.6% in Group B, and 75.0% in Group C. Regarding food categories, the beverage category (which included juices, hot beverages nutritional beverages and ready-to-drink beverages) had the highest percentage (28.8%) of Group A products compared to other categories (Supplementary Figure S1). There were no main meal and milk and dairy products that meet Group A criteria. Based on the marketing criteria, 96.4% of the 12,750 products did not meet the criteria for advertising to children. This included the 9.4% that are automatically prohibited from marketing, together with 5.0, 14.1, and 67.9% of products from Groups A, B, and C, respectively.

For *the WHO SEA model,* in a total sample of 17,030 products in 17 food categories, 88.1% did not meet the threshold for advertising to children. The food categories that had the highest percentage of products permitted for advertising were fresh and frozen fruits and vegetables and legumes (80.0%), fats and oils, and fat emulsions (64.5%), curded dairy-based desserts (34.3%), beverages (27.6%), and processed fruits and vegetables (27.2%). The classification of foods permitted and prohibited for advertising to children by food categories is shown in Supplementary Figure S2.

For t*he HCL model,* of a total sample of 12,282 products in 13 food categories, 88.0% did not meet the criteria to be eligible to display the HCL. Dairy products were mostly eligible for the HCL with 36.4% in this category, followed by instant foods (29.0%), seasonings (25.9%), ready-to-eat meals (23.6%), and ice creams (22.4%). The products that are eligible and not eligible to carry the front-of-pack HCL are shown in Supplementary Figure S3.

For *the NOVA system,* of the total sample of 17,058 products in 17 food categories (Mintel GNPD categories), 76.6% were classified as UPF, 14.6% as P, 6.1% as MP, and 2.8% as PCI. Categorizing by the Mintel GNPD category, fresh and frozen fruits and vegetables, and legumes were mostly classified as MP (80% of the total sample in this category), followed by solid-form soybean products (46.7%), processed fruits and vegetables (17.4%), beverages (12.5%), and cereals (7.7%) (Supplementary Figure S4). UPFs were found in all food categories especially desserts, confectionery, and composite foods.

### 3.3. Evaluation of healthy products based on one or more food classification systems

There was a total of 10,486 products (60.2% of the total sample) that could be assessed using all four different classification systems (Table 3). Of these, 67.7% (7,100 products) did not pass the criteria of any of the food classification systems (DOH, WHO SEA, HCL, and NOVA). The remaining products met at least one of the classification systems: 20.1% (2,111 products) met the “healthy” criteria of one system, 8.3% (874 products) met the criteria of two systems, 3.3% (344 products) met the criteria of three systems, and 0.5% (57 products) met the criteria of all four systems.

**Table 3:** Healthy products based on one or more food classification systems (*n* = 10,486).

Meeting the “healthy” criteria	Number of products	Percentage (%)
None	7,100	67.7
One system	2,111	20.1
Two systems	874	8.3
Three systems	344	3.3
Four systems	57	0.5

Of the 57 products meeting the healthy criteria for all four systems, 34 products were beverages (such as 100% palm juice, no sugar added instant ginger drink, and instant lemongrass drink), 20 were savory snack foods (such as natural baked almonds, roasted unsalted pistachios, and no salt roasted pumpkin kernels), and three were processed fruits and vegetables (such as roasted seaweed and rice cakes).

### 3.4. Comparison of the nutrient profiling-based systems with the food-processing-based system (NOVA)

Table 4 shows a summary of the classification results of the products (n = 10,486) that could be classified by any of the four classification systems. Of these, 83.1% were classified as UPF products and 16.9% as non-UPF products (combined MP, PCI, and P).

**Table 4:** Percentage of packaged products that can be classified by all four classification systems, in the 15 WHO categories (*n* = 10,486).

Category	NOVA				DOH			WHO SEA		HCL	
	MP	PCI	P	UPF	Group A	Group B	Group C	Marketing permitted	Marketing prohibited	Eligible	Not eligible
Confectionery	0.0	0.0	0.0	100.0	3.6	17.9	78.5	1.2	98.8	7.1	92.9
Fine bakery wares	0.2	0.0	11.2	88.6	2.2	7.6	90.2	0.2	99.8	1.5	98.5
Bread and ordinary bakery wares	0.0	0.0	32.6	67.4	1.3	7.2	91.5	1.6	98.4	6.9	93.1
Cereals	5.0	0.0	12.6	82.4	3.4	35.3	61.3	4.2	95.8	8.4	91.6
Ready-to-eat savories (savory snack foods)	4.9	0.0	17.9	77.2	9.7	20.0	70.3	4.0	96.0	8.4	91.6
Beverages	14.4	0.0	9.9	75.7	24.4	21.0	54.6	30.5	69.5	15.0	85.0
Frozen dairy based desserts and edible ices	0.7	0.0	8.2	91.1	1.9	43.9	54.2	0.9	99.1	15.3	84.7
Curded dairy based desserts	0.0	0.0	0.0	100.0	0.0	0.0	100.0	0.0	100.0	50.0	50.0
Composite foods (prepared foods)	0.4	0.0	6.5	93.1	0.4	1.4	98.2	1.7	98.3	18.2	81.8
Fats and oils, and fat emulsions	0.0	0.0	21.4	78.6	14.3	0.0	85.7	0.0	100.0	0.0	100.0
Pasta and noodles and like products	0.0	0.0	2.9	97.1	1.0	5.6	93.4	2.4	97.6	19.9	80.1
Processed meat, poultry, game, fish and fish products	1.6	0.0	16.4	82.0	11.1	39.7	49.2	1.1	98.9	3.7	96.3
Processed fruits and vegetables	8.5	0.0	39.4	52.1	19.7	33.8	46.5	12.7	87.3	28.2	71.8
Solid-form soybean products	0.0	0.0	100.0	0.0	100.0	0.0	0.0	0.0	100.0	0.0	100.0
Sauces dips, and dressings	0.0	0.0	19.0	81.0	14.3	0.0	85.7	0.0	100.0	9.5	90.5
Overall total (%)	5.6	0.0	11.3	83.1	10.4	15.6	74.0	11.1	88.9	10.9	89.1

DOH, the Department of Health; WHO SEA, the WHO South-East Asia Region; HCL, the Healthier Choice Logo; and NOVA, the food-processing-based food classification system; MP, unprocessed or minimally processed foods; PCI, processed culinary ingredients; P, processed foods; and UPF, ultra-processed food. Group A indicates healthy pass or meeting standard; Group B indicates not meeting standard; and Group C indicates far below standard.

**Figure 2:**
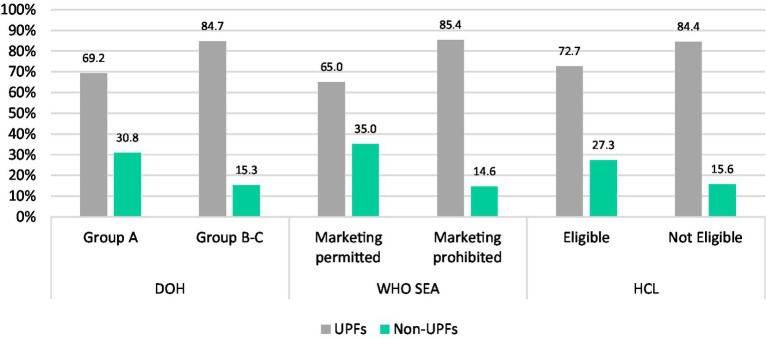
Percentage of healthy/healthier products based on nutrient profiling systems within NOVA categories.

Figure 2 shows the percentage of healthy products, which were classified as UPFs. Products that were eligible for the HCL had the highest proportion of UPF products (72.7%), followed by the Group A products (69.2%) and the “marketing permitted” products (65.0%). Meanwhile, 30.8, 35.0, and 27.3% of non-UPF products were classified as healthy/healthier products when evaluated using DOH, WHO SEA, and HCL systems, respectively.

### 3.5. Agreement scores

The average agreement between paired classification systems, ranged from 78.18% (HCL-NOVA) to 89.27% (DOH-WHO SEA) (Table 5). The kappa statistic for each pair of the four systems ranged from the poor agreement (0.09 in the HCL-NOVA) to moderate agreement (0.43 in the DOH-WHO SEA). There was poor agreement between NOVA and all of the other systems.

**Table 5:** Agreement scores (%) for food classification from the four different systems.

Food classification system	Agreement scores (%)					
	NOVA		WHO SEA		DOH	
	UPF	Non-UPF	Marketing prohibited	Marketing permitted	Group B-C	Group A
WHO SEA	*k* = 0.167 (79.79%)					
Marketing prohibited	7,961	1,358				
Marketing permitted	758	409				
DOH	*k* = 0.118 (79.31%)		*k* = 0.431 (89.27%)			
Group B-C	7,998	1,446	8,819	625		
Group A	721	321	500	542		
HCL	*k* = 0.094 (78.18%)		*k* = 0.212 (84.56%)		*k* = 0.210 (85.26%)	
Not eligible	7,889	1,455	8,522	822	8,621	723
Eligible	830	312	797	345	823	319

Values in the range of 0.01–0.20 are presented as “none to slight” agreement; “0.21–0.40” as fair agreement; “0.41–0.60” as moderate agreement; “0.61–0.80” as substantial agreement; and “0.81–1.00” as almost perfect agreement. DOH, the Department of Health; WHO SEA, the WHO South-East Asia Region; HCL, the Healthier Choice Logo; and NOVA, the food-processing-based food classification system; MP, unprocessed or minimally processed foods; PCI, processed culinary ingredients; P, processed foods; and UPF, ultra-processed food. Group A indicates healthy pass or meeting standard; Group B indicates not meeting standard; and Group C indicates far below standard.

## 4. Discussion

The study highlights three major findings. First, assessment of the nutrition quality based on different food classification systems yielded similar results and the majority of foods in each system were classified as unhealthy/not eligible. The data analyzed here suggest that the DOH, the WHO SEA, and the HCL nutrient profiling models distinguished their healthy or healthier options at almost the same percentage although they are somewhat different in terms of scope, application, nutrients to limit, and ingredients/nutrients to encourage.

Alignment between the DOH model and the WHO SEA model was confirmed with the highest agreement scores, which may be explained by the similar principles and rationale of these nutrient profile models as the DOH model was adapted from the WHO SEA model (24). Member States including Thailand were requested to comment on the WHO SEA model, and these comments were considered when finalizing the model (20). Despite similarities between the three models (DOH, WHO SEA, and HCL), each had differences in how healthy foods were classified, and thus having them operating simultaneously would be highly confusing when in one system a particular food would be healthy but in another would be unhealthy. This may lead to confusion for consumers, and also for policymakers and stakeholders regarding the application of these models in policy actions (15). For example, if the same food is assessed as healthy in one system but as unhealthy in another system, it would be confusing for those who need to use the system such as an advertiser who decides if they were permitted to advertise a product or not, and a school teacher who decides which foods to include on a menu. Ultimately, this could result in consumer confusion as they might see a product advertised but then that food is not permitted on the menu at school.

The second finding is that the criteria based on food processing yielded an even smaller number of healthy foods compared to the other systems. Less than 6% of new packaged food and beverage products released into the Thai marketplace were in their natural form or minimally processed, with no cosmetic additives. This result was almost two times lower than that of the DOH model which reported 10.4% for healthy products. This can be explained by differences in the principle and rationale of the food classification systems. Different from nutrient-based systems, the primary purpose of the NOVA is to classify based on the extent and purpose of industrial food processing (7).

By classifying foods based on processing, NOVA accounts for the nutrition quality of the whole food, including the way all biochemically active components of food interact, both with each other, and the food matrix in which they are contained [i.e., food synergy (29)]. This “food-based” classification challenges the dominant nutrient paradigm present in the nutrition discipline by contending that a food’s “healthfulness” is more than the sum of its nutrients (30). The mounting evidence on the association between UPFs and adverse health outcomes related to NCDs (4–6) necessitates a shift away from nutrient-only classification like that of the DOH model.

The third key finding is that the existing classification systems in Thailand do not capture UPFs, foods that are increasingly consumed by Thai people (13). A trend of UPF consumption is especially pronounced for ready-to-eat frozen foods and non-alcoholic sugar-sweetened beverages, which were consumed by 46.6 and 30.3% of the total Thai population at least 1–2 days per week, respectively (13). The market for these products also grew continually, with increasing sales of ready meals and soft drinks in Thailand at 10.7 and 2.1%, respectively, from 2018 to 2019 (14). Thailand also had one of the highest UPF annual sales growth rates in the world (31). Given the documented harms from the high consumption of UPFs (13), it is important to consider this in any system that classifies food to inform policy.

In addition to these challenges, the implementation of the 20-year National Strategy (2018–2037), which aims to turn Thailand into a developed country by 2037 (32), makes tackling UPF consumption in Thailand even more challenging. The strategy places priority focus on policies and strategies that can push Thailand to become “a developed country” in the next 20 years (32). Core government actions include “S-curve industries” which aims to speed up development and investment in targeted industries (including the food industry) and the “Food for the Future” initiative which aims to advance food and nutrition manufacturers with significant investment in food technology and innovation. Accordingly, food industries offering food innovation or production using modern or high-processing technology are welcomed with monetary and non-monetary incentives for domestic investment (33). Despite the economic benefits this type of strategy might bring, the diets of Thai people are likely to worsen further if food innovations result in increased UPF supply, which has implications for necessary increased healthcare expenditure in Thailand.

To the best of our knowledge, this is the first comparative evaluation of the nutrition quality of the food supply using various food classification systems in Thailand and Southeast Asia. This is also the first study analyzing the food supply using the WHO SEA model.

### 4.1. Policy implications

The evaluation of the nutrition quality of food products using different food classification systems in this study has several implications for future efforts to improve diets for better health outcomes in the Thai population. First, having two misaligned nutrient-based models operating simultaneously in Thailand may lead to confusion or misunderstanding among policymakers and stakeholders. Therefore, there is a need to combine them into one single model and as such can enhance their use in decision-making and policy processes.

Second, in order to effectively tackle unhealthy diets, the application of a food classification system that incorporates the NOVA concept (26, 34) is necessary to capture the nature of products being launched in the market, especially UPFs, which the existing models fail to do. A change from a solely nutrient profiling-based food classification system to a hybrid classification system that includes a food processing dimension may present some political and administrative challenges but will reflect good practice in translating the latest evidence. It has been shown that a holistic approach to nutrition classification, combined with a degree of processing, can help improve the existing nutrition classification model to classify the health potential of individual foods for policy purposes, preventing the promotion of ultra-processed foods (26, 34).

Third, the government should innovate for healthy food environments by promoting non-UPF products and meals in the markets and making them more available, affordable, and convenient. Food innovations should include “whole food reformulation or development of less processed alternatives (35),” rather than a nutrient reformulation of ultra-processed products. This can be achieved with agricultural and economic incentives in future such as applying subsidies on unprocessed and minimally processed foods. This may not only make the products more affordable for Thai consumers and promote the development of less processed products but also maintain the company’s profits and ultimately the country’s economy.

Fourth, there is a need for a strong global movement through international levers such as UN agencies and groups such as CODEX (36), which dictate food standards in trade negotiations throughout the world, for universal food-processing-based classification and UPF consumption reduction. Such schemes can also support government progress toward all sustainable development goals (SDGs), particularly SDG2 (Zero Hunger) and 3 (Good Health and Well-being) (37).

## 5. Limitations

There are some limitations of this study. The study relied on Mintel GNPD ingredients data that are retrieved mainly from modern retailers and thus may miss some food products which are locally made or sold in local and traditional stores. The study only included packaged products in the sample and some products were excluded from the analysis. Thus, the types of products included may have influenced the results because unpackaged foods typically are unprocessed and minimally processed foods. This study did not include store-bought unpackaged foods and beverages which are also commonly consumed (13) and baby foods, which are often UPFs (38). Despite these, the Mintel GNPD remains the largest data source that can provide detailed nutrition information and ingredients description of foods and beverages currently marketed in Thailand and, thus, facilitated the analysis in a systematic way.

## 6. Conclusion

This study assessed the nutrition quality of food and beverage products in Thailand based on four different food classification systems and compared nutrient profiling-based classification systems with a food-processing-based classification system and found large variations between the different systems. The results can help decision-making bodies, such as governments and nutrition associations, to determine the best approach for the nutrition classification of food and beverages for policy in Thailand. This will ensure a consistent approach that aids policies promoting healthy diets and achievement of SDGs and global NCD targets in this critical decade moving toward 2030.

The findings suggest that the optimal approach for nutrition classification is likely to be a combined assessment of processing with some nutrient of concern considerations. A holistic approach should be taken for the development and sustainable application of a food classification system in Thailand by taking both nutrients and food processing into account in order to fully capture unhealthy foods.

## Data availability statement

The original contributions presented in the study are included in the article/Supplementary material, further inquiries can be directed to the corresponding author.

## Author contributions

SP conceived the study, collected and analyzed the data, and developed and edited the manuscript. SD, NT, and ST collected and analyzed the data, and contributed to developing the study design and methods, and editing the manuscript. PM, JW, and ML advised on the study design and methods, supported the analysis, and provided significant guidance and editing to the draft manuscript. LM-S, PP, CU, and CK provided comments to the draft manuscript. All authors contributed to the article and approved the submitted version.

## Funding

This work was funded by the Thai Health Promotion Foundation (Grant number 64000197) and the Sweet Enough Network of Thailand. The funders had no role in the study design, data collection and analysis, decision to publish, or preparation of the manuscript.

## Conflict of interest

PP and CK declare that they have received salary from the Sweet Enough Network of Thailand. CU declares that she is a steering committee to the Thai Health Promotion Foundation.

The remaining authors declare that the research was conducted in the absence of any commercial or financial relationships that could be construed as a potential conflict of interest.

## Publisher’s note

All claims expressed in this article are solely those of the authors and do not necessarily represent those of their affiliated organizations, or those of the publisher, the editors and the reviewers. Any product that may be evaluated in this article, or claim that may be made by its manufacturer, is not guaranteed or endorsed by the publisher.
