# Supplementary material for: Choosing an effective food classification system for promoting healthy diets in Thailand: a comparative evaluation of three nutrient profiling-based food classification systems (government, WHO, and Healthier Choice Logo) and a food-processing-based food classification system (NOVA)
Source: Front Nutr. 2023 May 17;10:1149813. doi: 10.3389/fnut.2023.1149813 (PMC10230096; doi:10.3389/fnut.2023.1149813)
Supplement: Supplementary file 1 [file Data_Sheet_1.zip › FigureS3.docx]

Figure S3 Percentage of products eligible and not eligible for the HCL by food category

HCL - the Healthier Choice Logo
